# Alignment-Free Genome Geometry and Mosaic Barcodes Characterize Modular Diversity in the Cacao Swollen Shoot Virus Complex

**DOI:** 10.3390/v18090932

**Published:** 2026-08-25

**Authors:** Ezekiel Ahn, Insuck Baek, Jishnu Bhatt, Sookyung Oh, Minhyeok Cha, Lalit Kandpal, Seunghyun Lim, Moon S. Kim, Sunchung Park, Lyndel W. Meinhardt

**Affiliations:** 1 Sustainable Perennial Crops Laboratory, Agricultural Research Service, United States Department of Agriculture, Beltsville, MD 20705, USA; 2 Environmental Microbial and Food Safety Laboratory, Agricultural Research Service, United States Department of Agriculture, Beltsville, MD 20705, USA

**Keywords:** CSSV, *Badnavirus*, viral genomics, alignment-free, *k*-mer analysis, modular diversity

## Abstract

Cacao swollen shoot disease (CSSD) remains a major viral threat to cacao production in West Africa and is associated with a genetically diverse complex of badnaviruses. We combined alignment-free whole-genome distances with circular sliding-window mosaic barcodes to characterize global divergence and local compositional modularity across 48 full-length CSSD-associated badnavirus genomes, hereafter termed the cacao swollen shoot virus (CSSV) complex. Whole-genome tetranucleotide cosine distance clusters matched published species assignments exactly (adjusted Rand index = 1.00; normalized mutual information = 1.00; silhouette = 0.703) and were strongly concordant with an alignment-based distance derived from open reading frame 3 (ORF3) proteins (Spearman *ρ* ≈ 0.951; permutation *p* ≈ 0.0002). A three-component mosaic-complexity score summarized low dominant-label purity, barcode entropy normalized by log_2_(*K*), and circular switch rate; ORF–mosaic agreement was retained as a separate diagnostic and did not contribute to the ranking. Threshold sensitivity showed that barcode switchpoints were enriched within ±100 and ±200 bp of predicted ORF boundaries, but not within ±400 bp. The ten highest-scoring genomes showed slightly higher mean local nucleotide entropy but lower entropy variance and dispersion than the remaining genomes, indicating more uniformly distributed compositional complexity rather than isolated local spikes. These analyses provide a transparent framework for post-sequencing classification, comparison, and prioritization of complete viral genomes. The barcode and score outputs are exploratory summaries and are not direct field diagnostic assays or nucleotide-resolution recombination tests.

## 1. Introduction

Cacao (*Theobroma cacao* L.) is more than an agricultural commodity; it is the economic anchor of West Africa, supplying the vast majority of the global cocoa market. Yet, this stability is perpetually threatened by cacao swollen shoot disease (CSSD), which is driven by a genetically diverse assemblage of badnaviruses (family *Caulimoviridae*), hereafter referred to as the CSSV complex [1,2]. This pathogen complex poses a persistent operational challenge to eradication and replanting programs [3,4]. Despite decades of surveillance, the disease continues to expand, fueled by extensive genetic diversity, mixed infections, and interspecific recombination within the complex [2,5]. Consequently, the biological reality of CSSV is not a tidy lineage of distinct species, but a dynamic, reticulate evolutionary web where genomes merge and diverge [6].

This biological complexity presents a significant challenge for viral phylogenomics. Traditional taxonomy relies on multiple sequence alignment (MSA) to infer evolutionary histories. However, alignments presuppose collinearity and preservation of homologous structures, assumptions that arguably break down in badnaviruses, where recombination shuffles genomic modules and, more broadly, endogenous viral elements can complicate inference in badnavirus systems [2,7]. When genomes are shaped by modular exchange rather than descent with modification alone, a single phylogenetic tree based on alignment may oversimplify complex evolutionary histories [8]. Consequently, there is a critical need for an analytical framework that can capture the global structure of viral diversity without being constrained by the local artifacts of alignment algorithms.

Alignment-free methods, particularly those based on *k*-mer frequency distributions, offer a compelling alternative. By treating genomes as points in a high-dimensional feature space, alignment-free approaches provide a “genome geometry” that is robust to rearrangements and computationally scalable [9,10]. Recent studies have demonstrated the utility of alignment-free sequence representations in viral analysis, including host prediction for coronaviruses and influenza A viruses and whole-genome phylogenomics of coronaviruses [11,12]. Information-theoretic summaries can also be useful as descriptive measures of sequence heterogeneity. For example, Shannon entropy provides a compact way to summarize local compositional variability across sliding windows, while Jensen–Shannon distance provides a symmetric distance between *k*-mer probability distributions [13,14,15].

We asked two linked questions. First, can an alignment-free whole-genome distance representation preserve the major lineage structure recovered from a separately computed open reading frame 3 (ORF3)-based alignment? Second, what patterns of local compositional modularity are revealed by sliding-window *k*-mer barcodes, and how do those patterns relate to ORF-based clustering? To address these questions, we combined whole-genome *k*-mer geometry, sliding-window barcode summaries, ORF3-based distances, and descriptive information-theoretic measures. This design treats alignment-free and alignment-based representations as complementary views of the same dataset. We further used sliding-window Shannon entropy and Jensen–Shannon distance as descriptive summaries of local and genome-scale compositional variation. Together, these analyses provide a reproducible framework for characterizing genome-scale divergence and local modular heterogeneity in the CSSV complex.

## 2. Materials and Methods

### 2.1. Genome Dataset and Sequence Curation

We assembled a curated dataset of 48 full-length CSSD-associated badnavirus genomes from publicly available National Center for Biotechnology Information (NCBI) records. Forty-six genomes were retrieved using the NCBI nucleotide query “Cacao swollen shoot virus” [Organism] AND complete genome (accessed on 10 January 2026). Two additional full-length cacao-infecting badnavirus genomes (AJ608931.1 and MN179342.1) were retained by accession-level manual curation because their genome length, organization, and record metadata linked them to CSSD despite their absence from the organism-level query. Each accession was screened for the expected badnavirus genome length and organization and for record-level indicators of endogenous viral elements (EVEs), host-flanking sequence, or host-genome assembly origin. No retained record contained such indicators. This public-record screen does not experimentally establish episomal status. Published species assignments were compiled from Gonzalez Grande and Micheli [16] and International Committee on Taxonomy of Viruses (ICTV) designations [17] (Table 1; Supplementary Data S1).

Sequences were parsed in Python using Biopython SeqIO [18] from GenBank records or FASTA-format text sequence files. Accession-level identifiers were preserved for cross-linking among outputs. All genomes were analyzed as circular molecules: origin-spanning windows were extracted from circular sequence extensions, the final-to-first barcode boundary was included when counting label transitions, and positional comparisons used the minimum clockwise or counterclockwise distance.

### 2.2. Basic Sequence-Level Quality Control

For each genome, standard sequence-level summary statistics were computed to characterize the dataset and to screen for obvious anomalies. These included genome length, guanine–cytosine (GC) fraction computed over unambiguous bases only (A/C/G/T), the fraction of N characters, base counts (A, C, G, T, N), and Shannon entropy over A/C/G/T as a compact measure of compositional complexity [19]. These descriptive statistics were used to confirm expected genome size ranges, quantify ambiguity content, and provide baseline context for downstream alignment-free and information-theoretic analyses.

### 2.3. Alignment-Free Genome Geometry from k-mer Composition

To quantify whole-genome similarity without multiple sequence alignment (MSA), genomes were represented using *k*-mer frequency distributions, a standard alignment-free paradigm [10,20]. Tetranucleotide composition (*k* = 4) was used to balance resolution and statistical stability for these short viral genomes (≈7 kb), avoiding the sparsity of higher-dimensional vocabularies while retaining an established oligonucleotide signature. For each genome, all length-*k* substrings were extracted and counted; *k*-mers containing characters outside A, C, G, and T were discarded; and each vector was normalized by the number of retained *k*-mers. Each genome was represented in a 4*^k^*-dimensional feature space. Pairwise cosine distances yielded a symmetric genome-by-genome distance matrix.

This distance matrix defines an alignment-free “genome geometry”: each genome is a point in a metric space whose coordinates are not explicit nucleotide alignments but are implicit in the pairwise distances derived from compositional profiles. This representation is particularly useful for short circular genomes where alignment can be sensitive to local heterogeneity or indel-rich regions, and it enables fast and reproducible comparisons that scale naturally as additional genomes are added.

### 2.4. Ordination of Genome Distances by Metric Multidimensional Scaling

Metric multidimensional scaling (MDS) was applied to the whole-genome distance matrix to obtain a two-dimensional embedding [21]. MDS was implemented in scikit-learn [22] using the precomputed dissimilarity matrix. The two axes are arbitrary orthogonal coordinates selected to preserve pairwise dissimilarities and do not represent percentages of explained variance. Embedding quality was evaluated using Kruskal Stress-1 and the correlation between original and two-dimensional pairwise distances. For heatmap visualization, genomes were ordered by the first MDS coordinate.

### 2.5. External Validation Against Published Species Assignments

The whole-genome distance matrix was clustered by average-linkage agglomeration into three groups, matching the number of published species represented in the 48-genome panel. Cluster labels were compared with published assignments from Gonzalez Grande and Micheli [16] and ICTV nomenclature [17] using the adjusted Rand index (ARI), normalized mutual information (NMI), and silhouette width. Species labels and accession-level assignment sources are provided in Table 1 and Supplementary Data S1.

### 2.6. Sliding-Window Mosaic Barcodes for Local Modular Structure

Each circular genome was divided into overlapping 250 bp windows advanced in 50 bp steps, including windows spanning the sequence origin. Window *k*-mer vectors (*k* = 4) were computed with the whole-genome filtering rules and pooled across genomes. The baseline representation used *k*-means clustering with *K* = 8 and a fixed random state [22,23]. To evaluate this choice, candidate *K* values from 2 to 12 were assessed using silhouette width [24], Calinski–Harabasz and Davies–Bouldin indices, and cross-seed adjusted Rand index stability across 10 random seeds. Because no criterion identified a single universal optimum, *K* = 8 was retained as the pre-specified intermediate-resolution descriptive partition. As an alternative geometry check, Euclidean *k*-means assignments were compared with spherical *k*-means on L2-normalized vectors [25].

Each window inherited a discrete cluster label, and the ordered sequence of labels along the genome coordinate defined a “barcode” representing the local modular structure of that genome in the *k*-mer feature space. This barcode provides a compact, alignment-free representation of local sequence modules and their arrangement along the genome, enabling quantification of modular mixing and visualization of local mosaic patterns.

### 2.7. Switchpoint Definition and Per-Genome Mosaic Complexity Metrics

A switchpoint was defined as a boundary between consecutive windows with different barcode labels, including the final-to-first transition for circular genomes. For per-genome barcode summaries and mosaic-complexity scoring, circular switch count and switch rate were calculated from all adjacent label changes, with the number of circular window boundaries used as the denominator. For the ORF-boundary proximity analysis, switchpoint positions were assigned to the start coordinate of the first window in the new run, and only transitions separating runs of at least two consecutive windows on each side were retained. Per-genome summaries also included the dominant label, dominant-label fraction (“purity”), raw Shannon entropy of the label distribution in bits (log base 2), entropy normalized as *H*/log_2_(*K*), and the number of labels observed.

### 2.8. De Novo ORF Prediction on Circular Genomes

Because coding sequence (CDS) annotations can be inconsistent across public records, open reading frames (ORFs) were also predicted de novo from nucleotide sequences without relying on GenBank feature tables. A six-frame scan was performed across the forward strand and reverse complement. ORFs began at ATG and terminated at the first in-frame TAA, TAG, or TGA codon. ORFs shorter than 300 amino acids (terminal stop excluded) were discarded. For circular genomes, wrap-around ORFs were detected by scanning a doubled sequence and retaining spans no longer than one genome length.

Predicted ORFs were recorded with strand, coordinates (0-based half-open indexing), wrap status, and nucleotide/amino-acid lengths. Nucleotide sequences for predicted ORFs were extracted from circular genomes using the recorded coordinates; wrapped ORFs were extracted as a suffix-plus-prefix concatenation across the origin. Negative-strand ORFs were extracted as reverse complements. Amino-acid sequences were generated by translation with the removal of any terminal stop character.

### 2.9. Switchpoint Proximity to ORF Boundaries and Permutation-Based Null Model

For each switchpoint, circular distance to the nearest predicted ORF start or end was calculated. Enrichment was evaluated by preserving the observed number of switchpoints within each genome and sampling the same number of random positions uniformly along that genome in 2000 permutations. The enrichment direction was pre-specified as an excess of observed switchpoints near ORF boundaries. Thresholds of ±100, ±200, and ±400 bp were evaluated. For each threshold, the empirical one-sided *p*-value was calculated as Pr(null fraction ≥ observed fraction).

### 2.10. ORF3 Proxy Selection, Multiple Sequence Alignment, and ORF-Based Phylogenetic Distances

The longest predicted ORF from each genome was selected as an ORF3 candidate and compared with the annotated canonical badnavirus ORF3/polyprotein feature in the corresponding GenBank record. Predicted and annotated strand, coordinates, length, and overlap were recorded. Amino-acid sequences were aligned using MAFFT v7.505 [26], and columns with a gap fraction greater than 0.5 were removed. Pairwise identity was computed after excluding sites containing a gap in either sequence, and ORF3 distance was defined as 1 minus pairwise identity.

An unrooted neighbor-joining (NJ) tree was inferred from the ORF3 distance matrix [27]. No biological outgroup was designated; midpoint rooting was used only to orient the rectangular display. Branch support was assessed by resampling alignment columns with replacement for 1000 bootstrap replicates [28], rebuilding the identity-distance NJ tree for each replicate, and reporting support for reference-tree bipartitions. Support values of at least 70% were retained for display on major clades containing at least five tips.

### 2.11. Distance–Distance Concordance and Mantel-Style Permutation Test

To quantify concordance between alignment-free genome distances and alignment-based ORF distances, the upper-triangle vectors of the two distance matrices were compared using Spearman’s rank correlation. Statistical significance was assessed using a Mantel-style permutation procedure in which genome labels were randomly permuted in one distance matrix and the correlation recomputed across 5000 permutations [29]. Two-sided empirical *p*-values were reported as the fraction of permuted correlations with an absolute value greater than or equal to the observed correlation. This analysis tests whether two distinct representations of divergence (whole-genome alignment-free distances and ORF3 alignment-based distances) preserve the same relative ordering of genome pairs.

### 2.12. ORF–Mosaic Agreement and Mosaic-Complexity Scoring

Genomes were assigned to eight ORF clusters (baseline *K* = 8) by average-linkage agglomerative clustering of the ORF3 distance matrix. Mosaic structure was summarized independently by each genome’s dominant barcode label. Agreement between the two partitions was quantified using the ARI [30] and NMI [31]; majority-vote mapping of ORF clusters to dominant barcode labels was retained as a separate diagnostic. The ranking score used only barcode-derived components. For genome *i*, the mosaic-complexity score was defined as:

Score*_i_* = (1 − *P_i_*) + *E_i_* + *S_i_*, (1)

where *P_i_* is dominant-label purity, *E_i_* is barcode-label entropy normalized by log_2_(*K*), and *S_i_* is circular switch rate. Higher values therefore indicate lower purity, greater use of multiple barcode labels, and more frequent circular label transitions. ORF–mosaic mismatch was not included in the score and was reported separately. The score is an exploratory genome-level summary of local compositional complexity, not a breakpoint-level recombination test.

### 2.13. Score Audit and Parameter-Sensitivity Analyses

Score robustness was evaluated by recording the full distribution and perturbing the weights of the three barcode-derived components. Barcode-parameter sensitivity was assessed one factor at a time around the baseline configuration (*k* = 4, window = 250 bp, step = 50 bp, *K* = 8), including *k* = 3 or 5, windows of 200 or 300 bp, step = 100 bp, and barcode *K* values of 6, 7, 9, and 10. ORF-cluster *K* was varied separately from 6 to 10 as a diagnostic. ORF prediction and boundary analysis were repeated with minimum ORF lengths of 200, 300, and 400 amino acids. Outputs are provided in Supplementary Data S1.

### 2.14. Information-Theoretic Profiling: Entropy Landscapes and Jensen–Shannon Distances

To connect genome variation to information-theoretic descriptors, circular sliding-window Shannon entropy profiles were computed along each genome using a 200 bp window and a 50 bp step. Entropy was computed over A/C/G/T characters only to avoid inflating complexity due to ambiguous calls. These profiles define an “entropy landscape” that summarizes positional heterogeneity in local sequence complexity.

In parallel, Jensen–Shannon distance (JSD) between genomes was computed from fixed tetranucleotide probability vectors (*k* = 4; 4*^k^* states). JSD is symmetric and bounded and complements cosine distance by emphasizing redistribution of probability mass among *k*-mer states. JSD matrices were compared with cosine *k*-mer distances and ORF3 distances using the Spearman and permutation framework described above.

To test whether the highest-scoring genomes exhibit distinct entropy distribution properties, the ten highest-scoring genomes were compared with all other genomes using per-genome entropy summary metrics computed across windows. These metrics included mean entropy, entropy variance, and dispersion/tail measures (e.g., interquartile range, p95–p05, and max–median). Group differences were assessed using two-sided permutation tests with 10,000 permutations, and effect sizes were reported as Cohen’s *d*.

### 2.15. Reference-Panel Nearest-Neighbor Projection

To provide broader evolutionary and geographic context, we performed an auxiliary nearest-neighbor projection of the 48 core genomes onto an expanded reference panel of 87 cacao-infecting badnavirus genomes. This panel comprised the 86 genomes curated by Gonzalez Grande and Micheli [16], supplemented with the NCBI reference sequence NC_001574.1; the complete accession list for this panel is provided in Supplementary Data S1. Using the same alignment-free tetranucleotide (*k* = 4) representation used for genome geometry, we identified the closest reference neighbor for each core genome based on minimum cosine distance. Isolate designations and regional descriptors were captured from public record descriptions to serve as context labels. This projection was used for interpretability only and did not alter any core analyses.

### 2.16. Software and Reproducibility

All analyses were implemented in custom Python scripts using Python 3.10.19, NumPy, pandas, scikit-learn [22], and Biopython [18]. MSA was performed with MAFFT v7.505 [26]. Stochastic procedures used explicit random seeds. The analysis code is publicly available at https://github.com/EJSAHN/cssv-geom-mosaic (accessed on 22 August 2026); the version used for the analyses reported here corresponds to commit 35439d5bfa4d0a5f36cb523385ab04506bc68a30 (release v1.0.0).

## 3. Results

### 3.1. Genome-Scale Geometry Reveals Structured Lineages in CSSV Without Alignment

**Figure 1:**
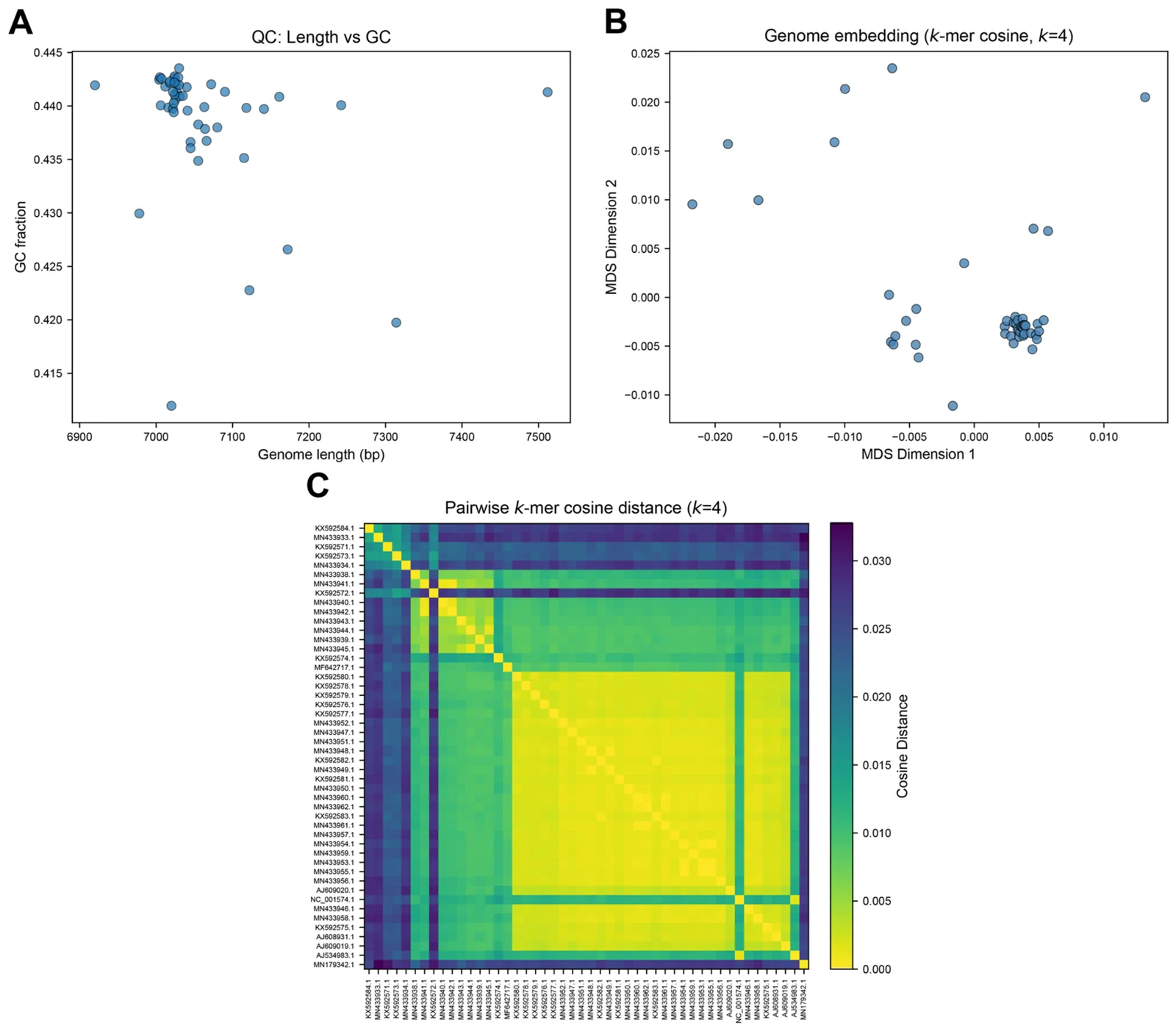
Alignment-free CSSV genome geometry from *k*-mer distances. (**A**) Genome length versus GC fraction for all 48 genomes. (**B**) Metric multidimensional scaling (MDS) embedding of the *k* = 4 cosine distance matrix. MDS1 and MDS2 are arbitrary coordinates chosen to preserve pairwise dissimilarities and do not represent explained variance; Kruskal Stress-1 = 0.171. (**C**) Whole-genome *k*-mer cosine distance heatmap.

We analyzed 48 full-length CSSD-associated badnavirus genomes (≈7 kb) using whole-genome tetranucleotide cosine distances and metric MDS. Genome length and GC fraction were relatively constrained (Figure 1A), whereas the MDS embedding resolved distinct groups and outliers (Figure 1B). The two-dimensional embedding had Kruskal Stress-1 = 0.171, and its reconstructed pairwise distances correlated strongly with the original matrix (Pearson *r* = 0.968). The distance heatmap showed coherent block structure (Figure 1C), demonstrating pronounced genome-scale organization despite the narrow quality control envelope.

### 3.2. Published Species Assignments Independently Validate Whole-Genome Clusters

Average-linkage clustering of the whole-genome *k*-mer distance matrix into three groups exactly reproduced the published species assignments for all 48 accessions: 41 cacao swollen shoot Togo B virus genomes, six cacao swollen shoot CE virus genomes, and one cacao swollen shoot Ghana T virus genome. Agreement was complete (ARI = 1.00; NMI = 1.00), with a silhouette score of 0.703 (Table 1; Supplementary Data S1).

### 3.3. Sliding-Window Mosaic Barcodes Quantify Within-Genome Modular Structure

**Figure 2:**
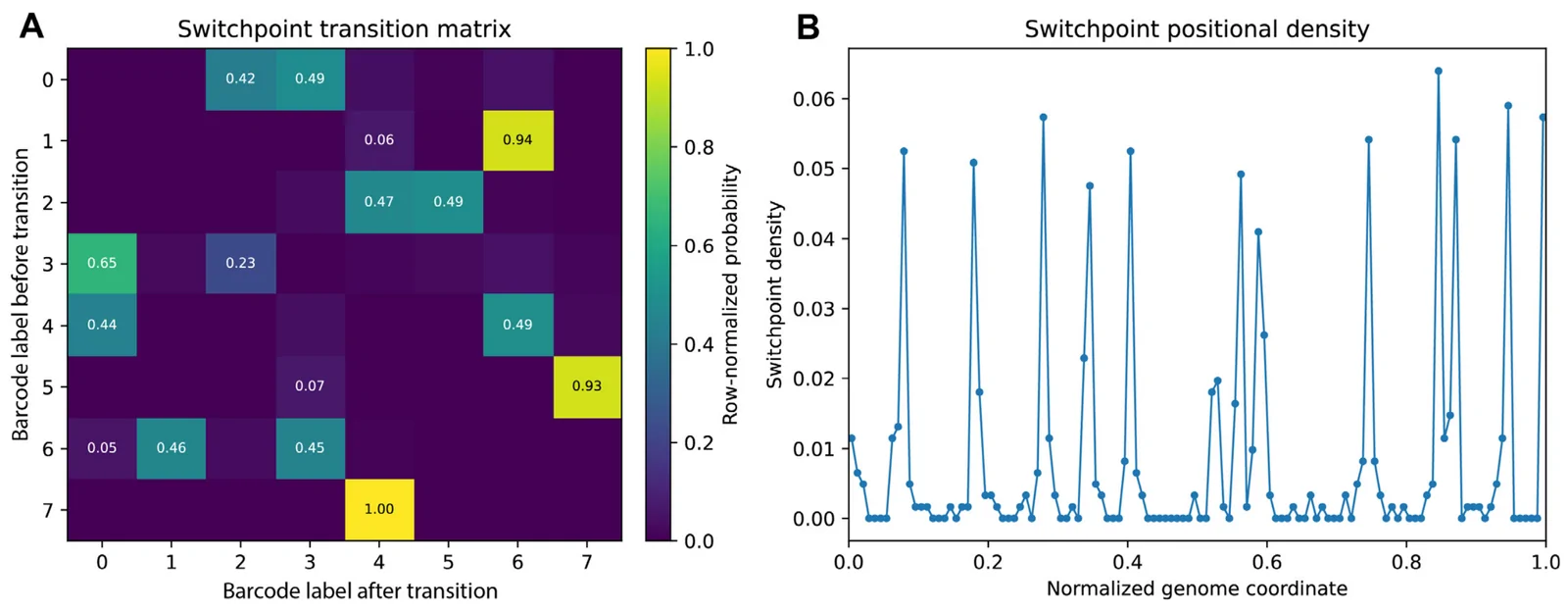
Mosaic barcode structure and switchpoint patterns across genomes. (**A**) Row-normalized transition matrix for circular barcode switches. Labels are displayed in numerical order (0–7) from the baseline *k*-means fit. Rows represent the barcode label before a transition, and columns represent the barcode label after the transition along the genome coordinate. Only non-zero entries are annotated. (**B**) Switchpoint density along the normalized circular genome coordinate.

Circular 250 bp windows advanced in 50 bp steps were clustered into *K* = 8 barcode labels. Genome barcodes ranged from profiles dominated by a few long blocks to profiles with more frequent switching among labels. The row-normalized transition matrix was sparse and non-uniform, and the normalized positional density contained multiple peaks rather than a flat profile (Figure 2). These summaries show structured local compositional variation but do not identify nucleotide-resolution recombination breakpoints.

### 3.4. Barcode-Clustering Diagnostics Define the Scope of K = 8

No single *K* value was optimal across all compactness and stability criteria evaluated over *K* = 2–12. Silhouette width and the Davies–Bouldin index favored *K* = 12, whereas the Calinski–Harabasz index and cross-seed stability favored *K* = 2. We therefore retained the pre-specified *K* = 8 setting as an intermediate-resolution descriptive partition rather than a data-derived optimum. At *K* = 8, Euclidean and spherical assignments showed partial agreement (ARI = 0.446; NMI = 0.612); circular switch rates remained correlated across geometries (Spearman *ρ* = 0.741). Complete diagnostics are shown in Supplementary Figure S1 and Supplementary Data S1.

### 3.5. Sequence-Only ORF Prediction Enables ORF-Boundary Proximity Tests Without GenBank CDS Annotations

**Figure 3:**
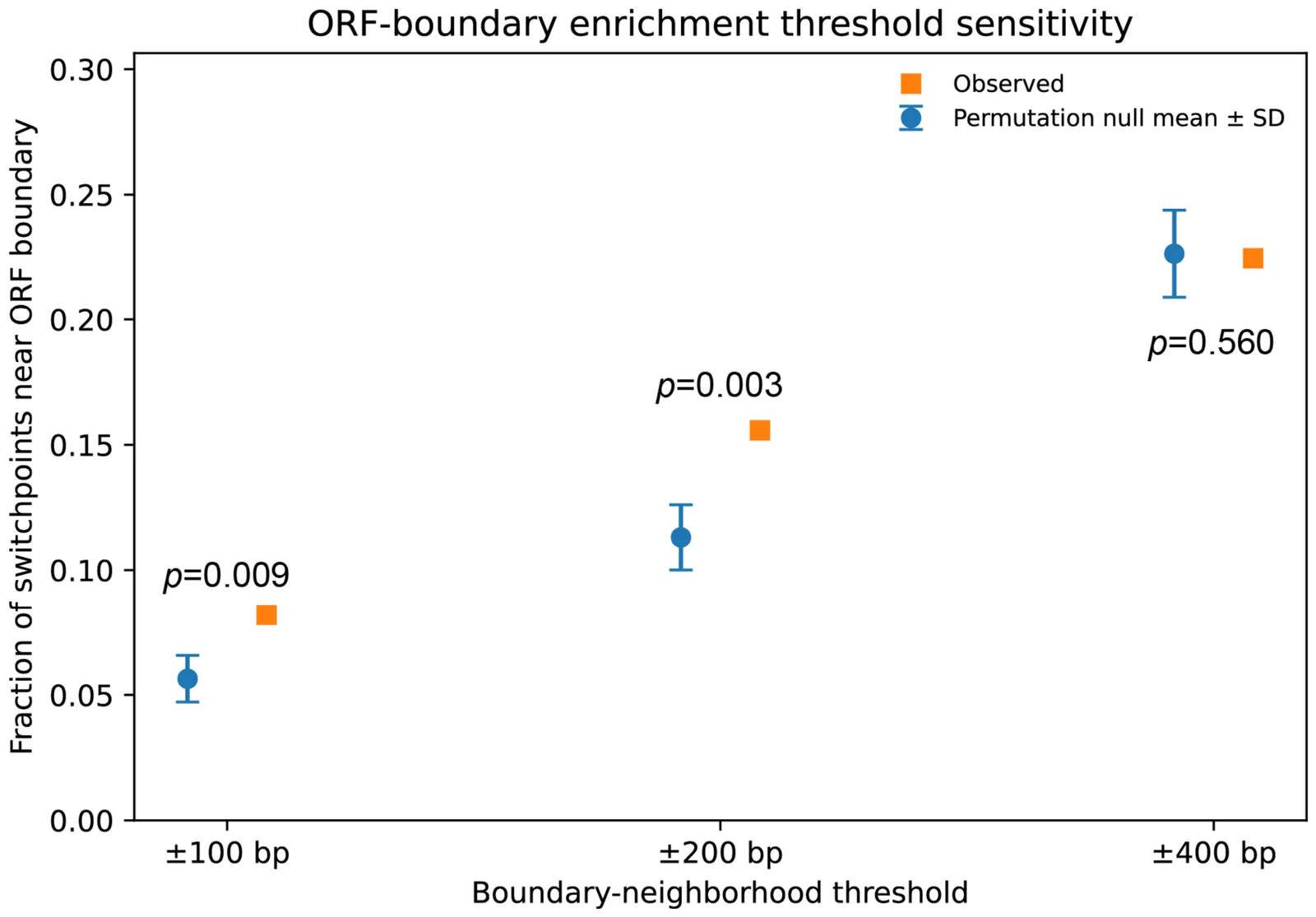
Sensitivity of switchpoint enrichment to ORF-boundary neighborhood size. Observed fractions of switchpoints within ±100, ±200, and ±400 bp of a predicted ORF boundary are compared with permutation-null means ± standard deviation (SD) from 2000 within-genome randomizations. *p*-values are one-sided and test the pre-specified enrichment direction. Enrichment was detected at ±100 and ±200 bp, but not at ±400 bp.

The circular barcode tracks yielded 610 filtered switchpoints. Switchpoints were enriched within narrow ORF-boundary neighborhoods: the observed fraction was 0.082 within ±100 bp versus a permutation-null mean of 0.056 (one-sided *p* = 0.009), and 0.156 within ±200 bp versus 0.113 under the null (*p* = 0.003). At ±400 bp, the observed fraction (0.225) was indistinguishable from the null mean (0.226; *p* = 0.560) (Figure 3). Thus, the association was confined to a narrow boundary scale rather than extending broadly across ORF-adjacent sequence. The complete distance distribution is shown in Supplementary Figure S2.

### 3.6. ORF3 (Longest ORF) Phylogeny Provides a Complementary, Alignment-Based View of Divergence

The longest predicted ORF corresponded to the annotated canonical badnavirus ORF3/polyprotein in all 48 genomes; no exceptions were detected in the accession-level audit (Table 1; Supplementary Data S1). ORF3 proteins were aligned with MAFFT, trimmed at a 0.5 gap-fraction threshold, and converted to pairwise identity-derived distances.

**Figure 4:**
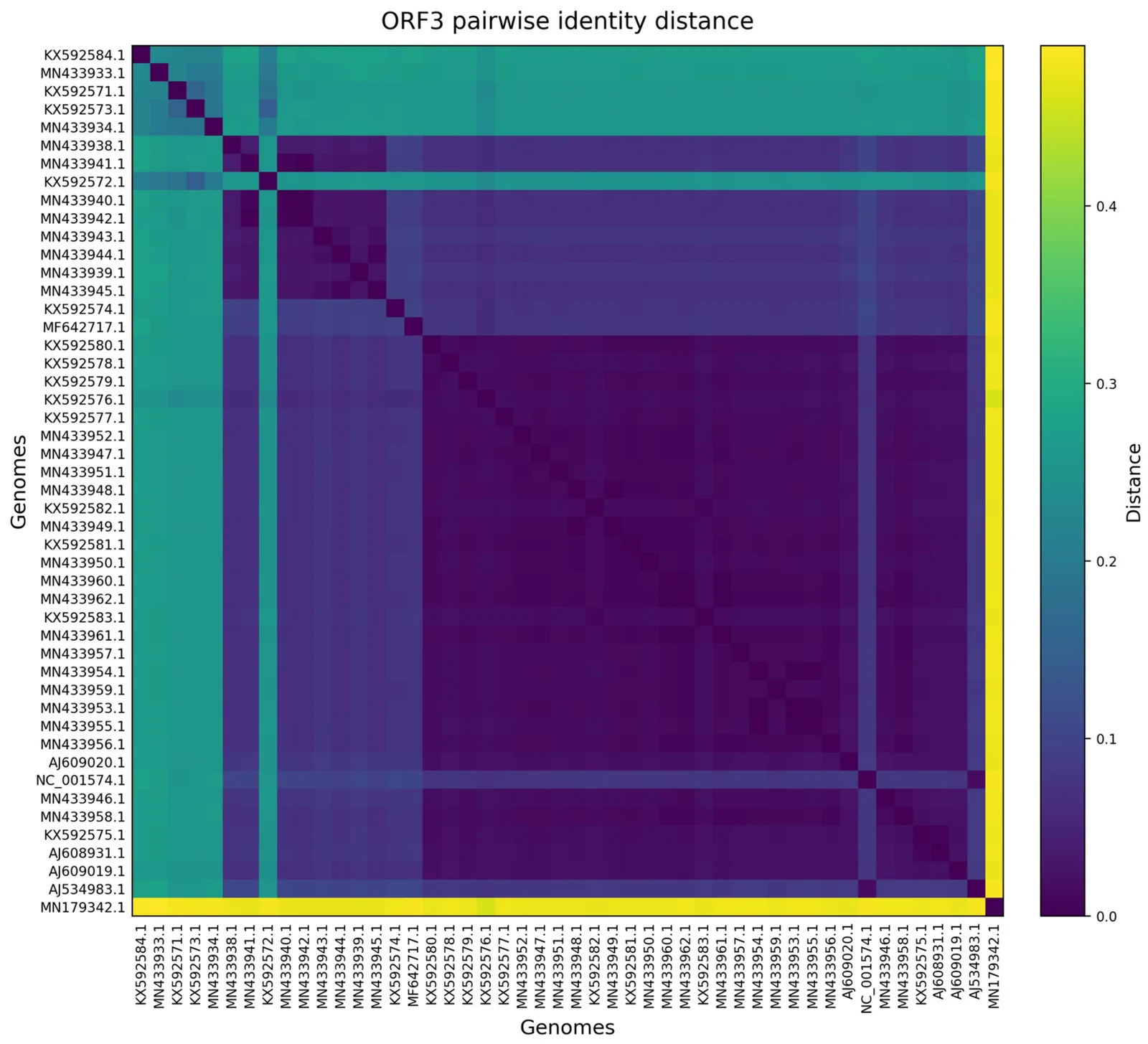
ORF-based distance structure.

The ORF3 distance matrix retained pronounced lineage-level block structure (Figure 4). The NJ topology was inferred without an outgroup; MN179342.1 was not treated as an outgroup, and midpoint rooting was used only to orient the rectangular tree display. Across 1000 bootstrap replicates, 25 of 45 non-trivial reference splits had support ≥70%, including 18 with support ≥90%.

Heatmap of ORF3 pairwise identity-derived distances (1 − identity) from the trimmed MAFFT alignment. Genomes are shown in the same order as in the whole-genome *k*-mer distance heatmap in Figure 1C to facilitate direct comparison of genome-scale and ORF3-based distance structure.

### 3.7. Genome-Scale k-mer Geometry Is Strongly Concordant with ORF3 Phylogenetic Distance

**Figure 5:**
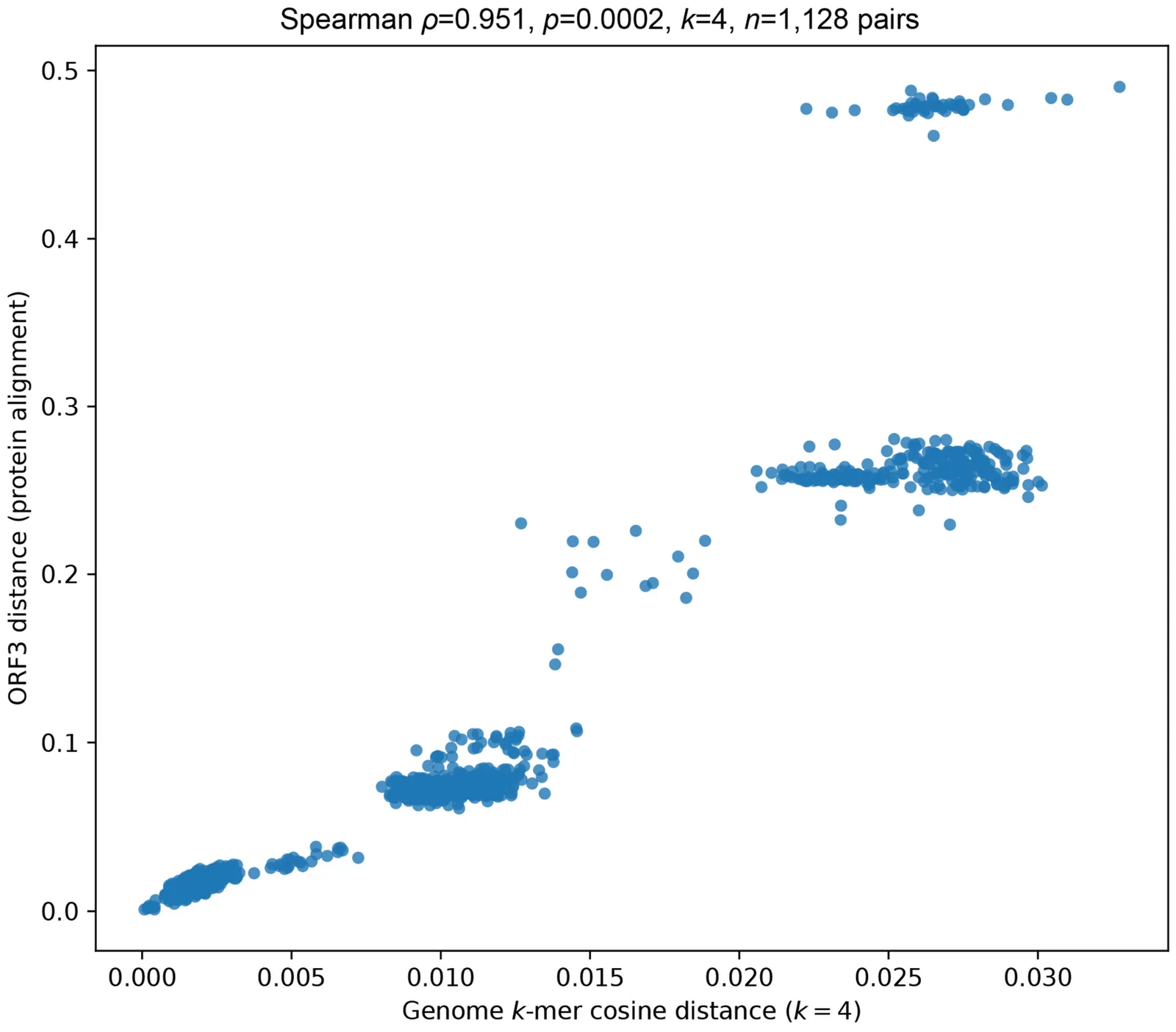
Concordance between alignment-free genome distances and ORF3 distances. Distance–distance scatter comparing *k* = 4 genome cosine distances (x-axis) to ORF3 identity distances (y-axis). Statistics are reported from a Mantel-style permutation test using Spearman correlation. The visual banding is consistent with a clustered structure.

Whole-genome tetranucleotide cosine distances and ORF3 identity-derived distances showed a strong monotonic association (Spearman *ρ* = 0.9507; two-sided permutation *p* = 0.0002; 48 genomes, 1128 pairs) (Figure 5). This concordance indicates that tetranucleotide resolution preserves the principal lineage-scale relationships represented in ORF3, while the external species comparison provides an independent validation of the whole-genome clusters.

### 3.8. A Mosaic-Complexity Score Summarizes a Continuous Range of Barcode Structure

**Figure 6:**
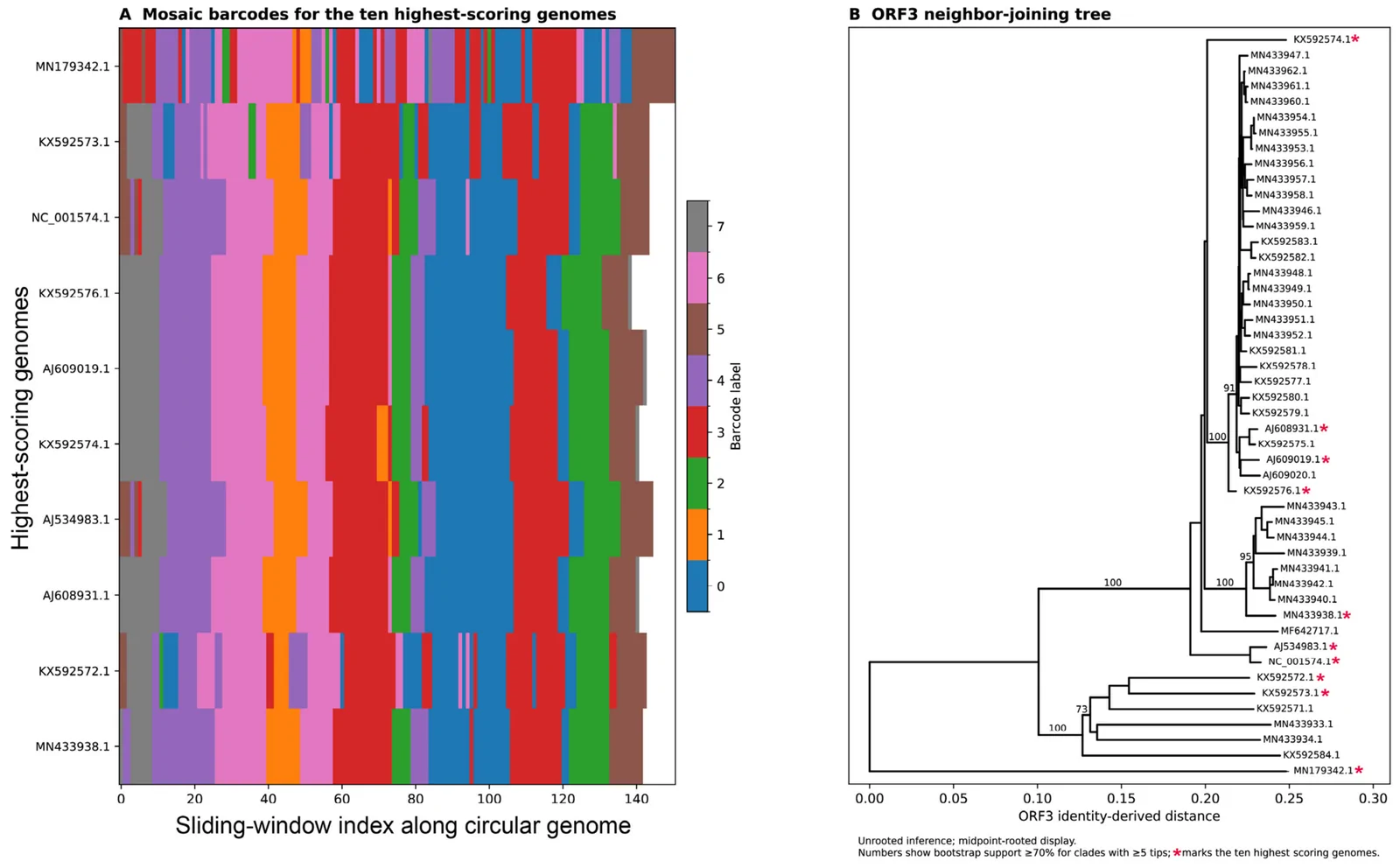
Highest-scoring genomes in mosaic barcode space and the ORF3 phylogeny. (**A**) Circular sliding-window barcode tracks for the ten highest-scoring genomes. (**B**) ORF3 neighbor-joining (NJ) tree inferred without an outgroup and midpoint-rooted for display only. Asterisks mark the ten highest-scoring genomes; numbers show bootstrap support ≥70% for major clades containing at least five tips, based on 1000 alignment-column replicates.

Each genome was ranked using the three-component mosaic-complexity score, which combines low dominant-label purity, normalized barcode entropy, and circular switch rate. Scores ranged continuously rather than forming a discrete class. The ten highest-scoring genomes had purity values of 0.194–0.285, raw barcode entropy of 2.518–2.879 bits, circular switch rates of 0.098–0.338, and composite scores of 1.847–1.892 (Table 2). Majority-vote ORF–mosaic mismatch was absent for all 48 genomes and therefore provided no ranking information; it was retained only as a separate diagnostic. Figure 6 places the highest-scoring barcodes beside the ORF3 tree.

### 3.9. Score and Clustering Sensitivity Define the Scope of the Baseline Ranking

The score distribution supported interpretation as a continuum: the rank-10 and rank-11 scores differed by only 2.24 × 10^−5^. Weight perturbations showed that different components emphasize different aspects of barcode structure, and no hard biological threshold was inferred. Barcode-parameter changes produced greater rank variation than ORF-cluster *K*, so the top 10 is reported as the baseline descriptive subset rather than a parameter-invariant class. Minimum ORF thresholds of 200, 300, and 400 amino acids recovered the same long-ORF set and did not alter the ORF3 audit conclusion.

To contextualize outliers and the highest-scoring genomes, we recorded the nearest match for each core genome in the expanded 87-genome reference panel (Supplementary Data S1). This projection adds interpretability by linking numerical rankings to isolate-style nomenclature and regional keywords available in public records. Recurring West African isolate designations and regional descriptors (e.g., ‘Buyo’, ‘Kipi’, ‘Krag’, and ‘Gha’ series; Supplementary Data S1) were recovered among nearest-reference matches. In addition, the NCBI reference sequence NC_001574.1 was recovered as a distance-zero match to L14546.1 within the panel, providing internal validation that the alignment-free metric recovers identical sequence records.

### 3.10. Highest-Scoring Genomes Show Reduced Entropy Dispersion Without Elevated Tail Extremes

**Figure 7:**
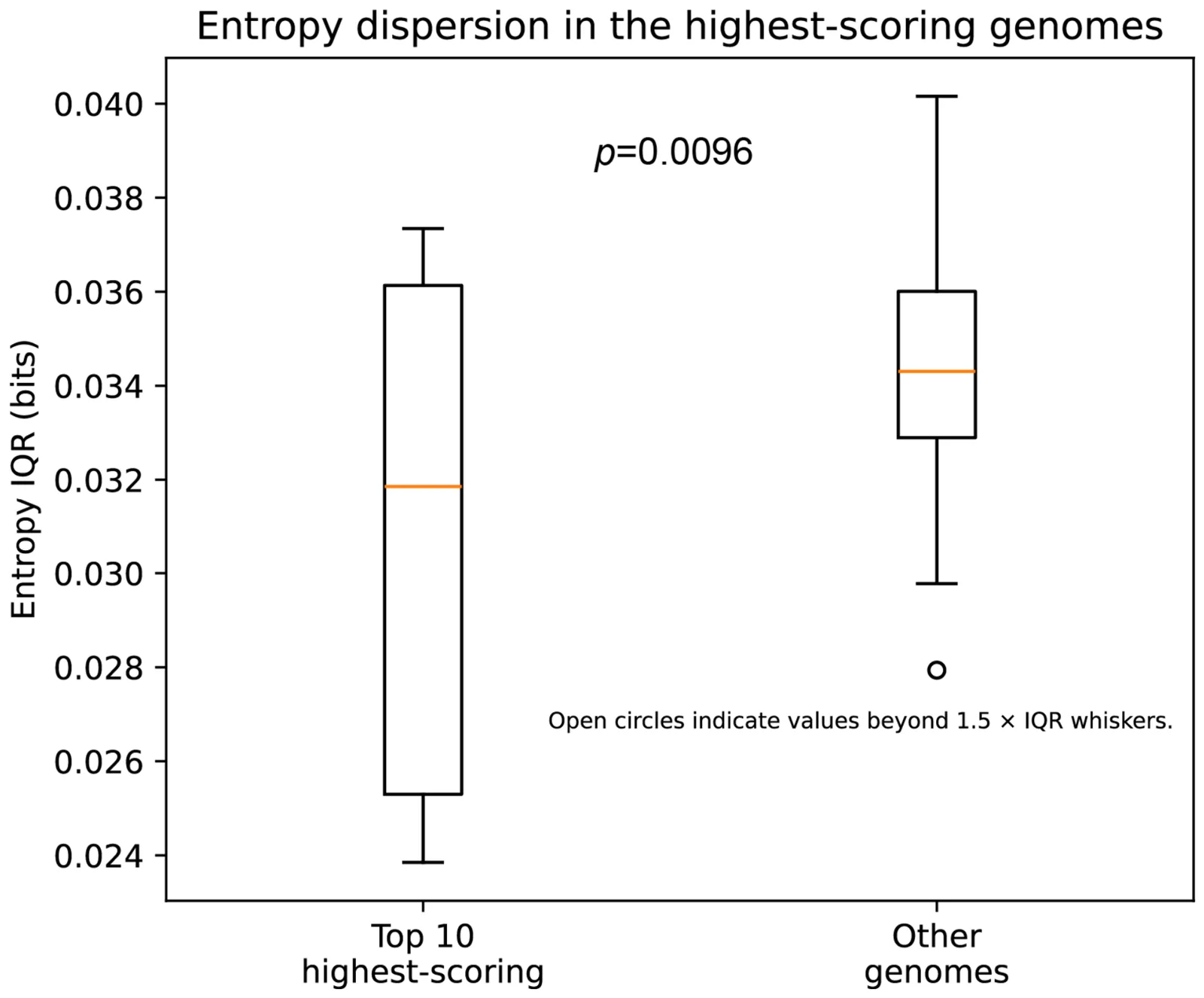
Entropy dispersion in the highest-scoring genomes. Boxplots compare the per-genome entropy interquartile range (IQR) between the ten highest-scoring genomes and the remaining genomes. The orange horizontal lines indicate the medians. The *p*-value is from a two-sided permutation test. Open circles indicate individual genomes lying beyond 1.5 times the interquartile range from the corresponding quartile. Additional mean, variance, dispersion, and tail metrics are reported in Supplementary Data S1.

The ten highest-scoring genomes had slightly higher mean local nucleotide entropy than the remaining genomes (difference = 0.00176; permutation *p* = 0.0003; Cohen’s *d* = 1.18) but lower entropy variance (*p* = 0.017; *d* = −0.88), interquartile range (IQR; *p* = 0.0096; *d* = −0.99), and p95–p05 spread (*p* = 0.0116; *d* = −0.92). Tail-extreme metrics were not elevated (p95, *p* = 0.571; max–median, *p* = 0.853; p95–median, *p* = 0.586). Thus, higher-scoring genomes showed more uniformly distributed compositional complexity rather than isolated entropy spikes (Figure 7; Supplementary Figure S3; Supplementary Data S1).

### 3.11. Entropy-Based Distances and Entropy-Dispersion Patterns

Both cosine distance and JSD captured a near-identical genome-scale structure. JSD between *k* = 4 *k*-mer probability distributions was strongly correlated with both *k*-mer cosine distances (Spearman *ρ* = 0.984; permutation *p* = 0.0002) and ORF3 alignment-based distances (Spearman *ρ* = 0.960; permutation *p* = 0.0002), indicating that the major lineage geometry is robust to the choice of alignment-free distance. Entropy distribution analyses provided an additional descriptive layer: the ten highest-scoring genomes did not show elevated tail extremes but showed reduced entropy dispersion relative to other genomes.

## 4. Discussion

### 4.1. Alignment-Free Genome Distances Preserve Major Lineage Structure

The whole-genome alignment-free representation was supported by two complementary validations. First, *k*-mer clusters matched published species assignments exactly across all 48 genomes (ARI = 1.00; NMI = 1.00; silhouette = 0.703). Second, pairwise whole-genome distances were strongly concordant with the ORF3 protein-distance representation derived from a separate alignment (Spearman *ρ* ≈ 0.95). These results show that the geometry is not explained solely by GC content or a two-dimensional visualization artifact and that it preserves recognized lineage-scale structure.

### 4.2. Published Species Assignments and ORF3 Support Biological Relevance

The species-level benchmark addresses an important distinction between internal concordance and external biological validation. The three whole-genome clusters recovered the 41 cacao swollen shoot Togo B virus, six cacao swollen shoot CE virus, and one cacao swollen shoot Ghana T virus assignments without disagreement [16,17]. The ORF3 comparison then showed that pairwise divergence ranks are also retained within the same sequence panel. Together, these comparisons support the use of the *k*-mer geometry for reference placement and unusual-sequence detection while leaving formal taxonomy to ICTV criteria and broader biological evidence.

The MDS axes themselves are arbitrary and should not be interpreted as biological variables. Their value is to visualize the distance matrix, whose two-dimensional reconstruction remained strong (Pearson *r* = 0.968). The barcode analysis addresses a different scale: local label diversity and switching within individual genomes. Global species placement and local barcode complexity should therefore be interpreted as complementary rather than interchangeable summaries.

### 4.3. Mosaic Barcodes Provide Quantitative Summaries of Local Modularity

Badnaviruses are widely discussed as evolutionarily plastic: mixed infections and recombination can generate new variants, and endogenous viral elements complicate diagnostics and taxonomy [2,32,33,34]. Our sliding-window mosaic barcode is intentionally agnostic about mechanism and does not claim to identify classical recombination breakpoints. Instead, each genome is represented as an ordered sequence of locally coherent *k*-mer modules, yielding three comparable summaries: dominant-label purity, mosaic-label entropy, and switch rate.

This intermediate representation provides genome-level summaries of local compositional heterogeneity that complement breakpoint-oriented recombination analyses. Barcode-derived metrics quantify the extent and spatial organization of label switching and support comparison among complete genomes (Table 2; Figure 6). The three-component score does not use ORF–mosaic mismatch, which was absent under the baseline mapping, and does not identify definitive recombination breakpoints. Established breakpoint-, sliding-window-, and similarity-based analyses remain appropriate for testing specific recombination hypotheses [35,36,37,38].

### 4.4. Fine-Scale Association of Barcode Switchpoints with ORF Boundaries

Barcode switchpoints were enriched within ±100 and ±200 bp of predicted ORF boundaries, whereas no enrichment was detected within the broader ±400 bp neighborhood. The scale dependence argues against a diffuse association across ORF-adjacent sequence and instead identifies a narrow boundary-associated compositional transition pattern.

Badnavirus recombination patterns can reflect both mechanistic exchange and selection against genomes that disrupt coding or protein-interaction constraints [2,39]. The observed fine-scale association is therefore compatible with modular organization or preferential retention of compositionally compatible exchanges near gene boundaries. However, barcode switchpoints are defined from overlapping 250 bp windows and are not nucleotide-resolution recombination breakpoints. The pattern should be tested with explicit breakpoint methods and domain-aware analyses of ORF3.

### 4.5. Relationship to Phylogeny and Recombination

The ORF3 NJ tree is an unrooted distance summary, not a complete reticulate history. Bootstrap resampling provides support for major bipartitions, but a bifurcating tree remains an approximation for a virus complex with documented recombination [2,32,34]. The tree is therefore used as a lineage reference axis rather than as evidence that all genomic regions share one history.

Reassortment in the strict sense was not evaluated because badnaviruses have single, non-segmented circular double-stranded DNA genomes [17]; there are no genome segments to exchange between strains. Recombination, rather than reassortment, is the relevant exchange process in this system. The barcode framework can identify genomes with unusual local compositional patterns, but parental strains and definitive recombinant events require explicit recombination analyses.

### 4.6. Practical Implications for CSSV Surveillance and Diagnostics

For surveillance, the framework is most directly applicable after complete or near-complete viral genomes have been recovered from field samples. A new sequence can be placed in the whole-genome distance geometry, compared with published species assignments and nearest reference genomes, and summarized by its barcode and mosaic-complexity score. Exact recovery of published species groups in the present panel supports rapid reference placement, while unusual geometry or barcode complexity can prioritize sequences for confirmatory recombination analysis, assay evaluation, or intensified sampling.

This is a post-sequencing genomic surveillance layer rather than a direct field diagnostic assay. Spatiotemporal tracking would require independently curated collection dates, locations, hosts, symptoms, and vector information. Mixed infections would additionally require haplotype-resolved assemblies or clone-level sequences because a single consensus genome can merge variants. High mosaic-complexity scores should not be equated with epidemiological risk, virulence, or recombinant fitness without field and experimental evidence. Used within these limits, the framework can support variant differentiation and prospective monitoring of circulating CSSV populations [6,40].

### 4.7. Entropy Dispersion as a Hypothesis-Generating Descriptor

The entropy results distinguish average local complexity from its variation along the genome. The highest-scoring genomes had slightly higher mean local nucleotide entropy but lower variance, IQR, and p95–p05 spread, with no increase in tail extremes. This combination indicates that compositional complexity was distributed more uniformly across the genome rather than concentrated in isolated high-entropy windows. This reduced dispersion could be consistent with genome-wide compositional constraint or selective persistence of compatible modular combinations. It is not, however, a direct measure of replication stability or recombinant fitness; those hypotheses require longitudinal persistence data and experimental infection or replication assays.

### 4.8. Limitations and Next Steps

Several limitations define the scope of inference. Record-level screening excluded sequences with EVE-related, host-flanking, or host-assembly indicators but did not experimentally establish episomal status. No single *K* value was optimal across all clustering criteria; *K* = 8 was retained as a pre-specified intermediate-resolution representation, and Euclidean and spherical clustering showed only partial assignment agreement. Genome rankings were sensitive to barcode parameterization and form a continuum without a natural top-10 threshold. NJ bootstrap values quantify support under alignment-column resampling but do not replace model-based phylogenetic or network inference. Finally, post-sequencing surveillance requires representative sampling and metadata that were not uniformly available for the public records analyzed here.

Priority next steps are explicit breakpoint and network analyses of high-scoring genomes [35,36,37,38], domain-aware testing within the ORF3 polyprotein, prospective application to dated and georeferenced field collections, haplotype-resolved analysis of mixed infections, and experimental evaluation of whether entropy-dispersion patterns relate to persistence or fitness.

To place the 48 core genomes in the broader context provided by the 86-genome analysis of Gonzalez Grande and Micheli [16], we projected them onto an 87-genome reference panel in the same alignment-free *k*-mer distance space (Supplementary Data S1). Because the associated labels are limited to information explicitly available in public records, we treat them as contextual descriptors rather than definitive sampling provenance.

## 5. Conclusions

Whole-genome tetranucleotide geometry recovered published species structure and closely tracked ORF3 divergence, while circular sliding-window barcodes quantified a distinct layer of local compositional modularity. Fine-scale enrichment of barcode switches near ORF boundaries and the more uniform entropy landscapes of the highest-scoring genomes generate specific hypotheses for follow-up without constituting breakpoint or fitness evidence. The framework provides a reproducible post-sequencing layer for comparative classification, unusual-variant prioritization, and integration with future spatiotemporal CSSV surveillance.

## Figures and Tables

**Table 1:** Accession-level dataset composition and published species assignments. Species acronyms: CSSTBV, cacao swollen shoot Togo B virus; CSSCEV, cacao swollen shoot CE virus; CSSGTV, cacao swollen shoot Ghana T virus [16,17].

Accession	Species	Route	Accession	Species	Route
AJ534983.1	CSSTBV	Query	MN433940.1	CSSTBV	Query
AJ608931.1	CSSTBV	Manual	MN433941.1	CSSTBV	Query
AJ609019.1	CSSTBV	Query	MN433942.1	CSSTBV	Query
AJ609020.1	CSSTBV	Query	MN433943.1	CSSTBV	Query
KX592571.1	CSSCEV	Query	MN433944.1	CSSTBV	Query
KX592572.1	CSSCEV	Query	MN433945.1	CSSTBV	Query
KX592573.1	CSSCEV	Query	MN433946.1	CSSTBV	Query
KX592574.1	CSSTBV	Query	MN433947.1	CSSTBV	Query
KX592575.1	CSSTBV	Query	MN433948.1	CSSTBV	Query
KX592576.1	CSSTBV	Query	MN433949.1	CSSTBV	Query
KX592577.1	CSSTBV	Query	MN433950.1	CSSTBV	Query
KX592578.1	CSSTBV	Query	MN433951.1	CSSTBV	Query
KX592579.1	CSSTBV	Query	MN433952.1	CSSTBV	Query
KX592580.1	CSSTBV	Query	MN433953.1	CSSTBV	Query
KX592581.1	CSSTBV	Query	MN433954.1	CSSTBV	Query
KX592582.1	CSSTBV	Query	MN433955.1	CSSTBV	Query
KX592583.1	CSSTBV	Query	MN433956.1	CSSTBV	Query
KX592584.1	CSSCEV	Query	MN433957.1	CSSTBV	Query
MF642717.1	CSSTBV	Query	MN433958.1	CSSTBV	Query
MN179342.1	CSSGTV	Manual	MN433959.1	CSSTBV	Query
MN433933.1	CSSCEV	Query	MN433960.1	CSSTBV	Query
MN433934.1	CSSCEV	Query	MN433961.1	CSSTBV	Query
MN433938.1	CSSTBV	Query	MN433962.1	CSSTBV	Query
MN433939.1	CSSTBV	Query	NC_001574.1	CSSTBV	Query

Query = NCBI organism-level complete-genome query; Manual = accession-level curation. Species acronyms follow published/ICTV assignments [16,17]. All 48 records passed the accession-level viral-record screen, and the longest predicted ORF matched the annotated ORF3/polyprotein in all 48 genomes. Record screening did not experimentally determine episomal status.

**Table 2:** Ten highest-scoring genomes by mosaic-complexity score. Raw barcode entropy is in bits; normalized entropy is *H*/log_2_(*K*), with *K* = 8. The score is (1 − purity) + normalized entropy + circular switch rate.

Rank	Accession	Mosaic-Complexity Score	Purity	Barcode Entropy (Bits)	Normalized Entropy	Circular Switch Rate
1	MN179342.1	1.892212	0.284768	2.517696	0.839232	0.337748
2	KX592573.1	1.867036	0.263889	2.788608	0.929536	0.201389
3	NC_001574.1	1.866669	0.222222	2.850006	0.950002	0.138889
4	KX592576.1	1.862449	0.194245	2.867923	0.955974	0.100719
5	AJ609019.1	1.861766	0.195804	2.879005	0.959668	0.097902
6	KX592574.1	1.858440	0.212766	2.873192	0.957731	0.113475
7	AJ534983.1	1.855525	0.227586	2.856230	0.952077	0.131034
8	AJ608931.1	1.854352	0.198582	2.860927	0.953642	0.099291
9	KX592572.1	1.850657	0.258741	2.677846	0.892615	0.216783
10	MN433938.1	1.847068	0.218310	2.836978	0.945659	0.119718

## Data Availability

The complete Python code used to run the analyses is publicly available at https://github.com/EJSAHN/cssv-geom-mosaic (accessed on 22 August 2026). The code state used here corresponds to commit 35439d5bfa4d0a5f36cb523385ab04506bc68a30. All viral genome sequences are available in GenBank, and accession lists and published species assignments for the core 48-genome dataset and the expanded reference panel are provided in Supplementary Data S1. The repository documents input preparation and reproduction of the analysis from public records.

## Supplementary Materials

The following supporting information can be downloaded at https://www.mdpi.com/article/10.3390/v18090932/s1. Supplementary Data S1 contains accession-level inclusion and species assignments, per-genome quality control metrics, whole-genome and ORF3 distance matrices, circular barcode assignments and switchpoint summaries, the three-component mosaic-complexity score and sensitivity outputs, external species validation, MDS diagnostics, clustering diagnostics, ORF3 bootstrap support, ORF-boundary threshold sensitivity, ORF3 annotation and EVE-record audits, entropy and JSD analyses, and nearest-reference projection tables. Figure S1: Barcode-clustering diagnostics across candidate *K* values; Figure S2: Distribution of switchpoint distances to predicted open reading frame (ORF) boundaries; Figure S3: Genome-wide entropy landscapes across cacao swollen shoot virus complex genomes.
