# Supplementary material for: Alignment-Free Genome Geometry and Mosaic Barcodes Characterize Modular Diversity in the Cacao Swollen Shoot Virus Complex
Source: Viruses. 2026 Aug 25;18(9):932. doi: 10.3390/v18090932 (PMC13612066; doi:10.3390/v18090932)
Supplement: Supplementary file 1 [file viruses-18-00932-s001.zip › viruses-4480327-supplementary.pdf]

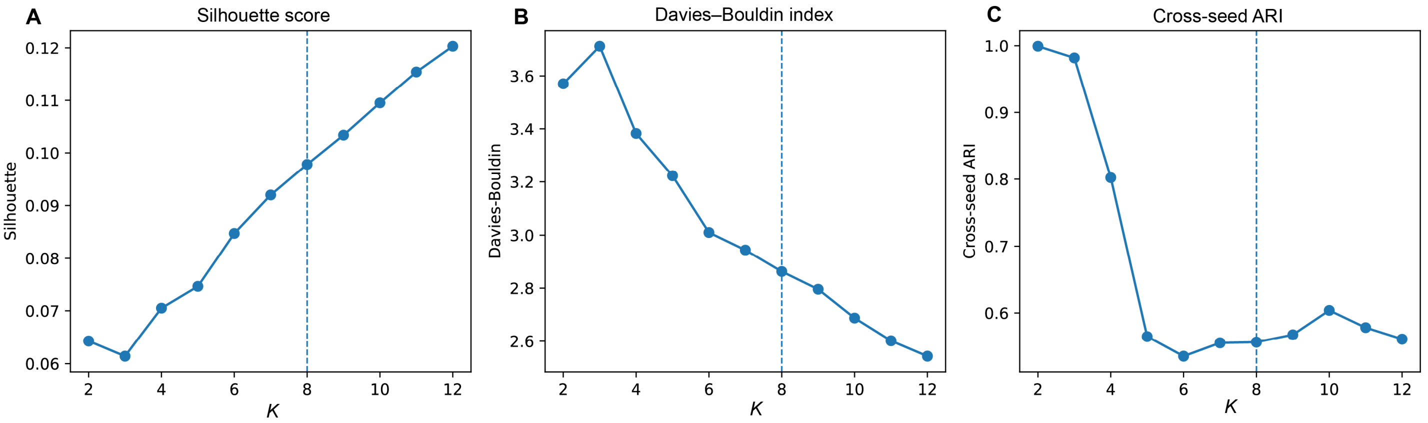

Figure S1. Barcode-clustering diagnostics across candidate *K* values.

(A) Mean silhouette score, for which higher values indicate greater separation. (B) Mean Davies–Bouldin index, for which lower values indicate better compactness and separation. (C) Mean pairwise adjusted Rand index across random seeds, used as an assignment-stability measure. No single K value was optimal across all criteria; $K = 8$ was retained as an intermediate-resolution descriptive partition, with full diagnostics reported in Supplementary Data S1.

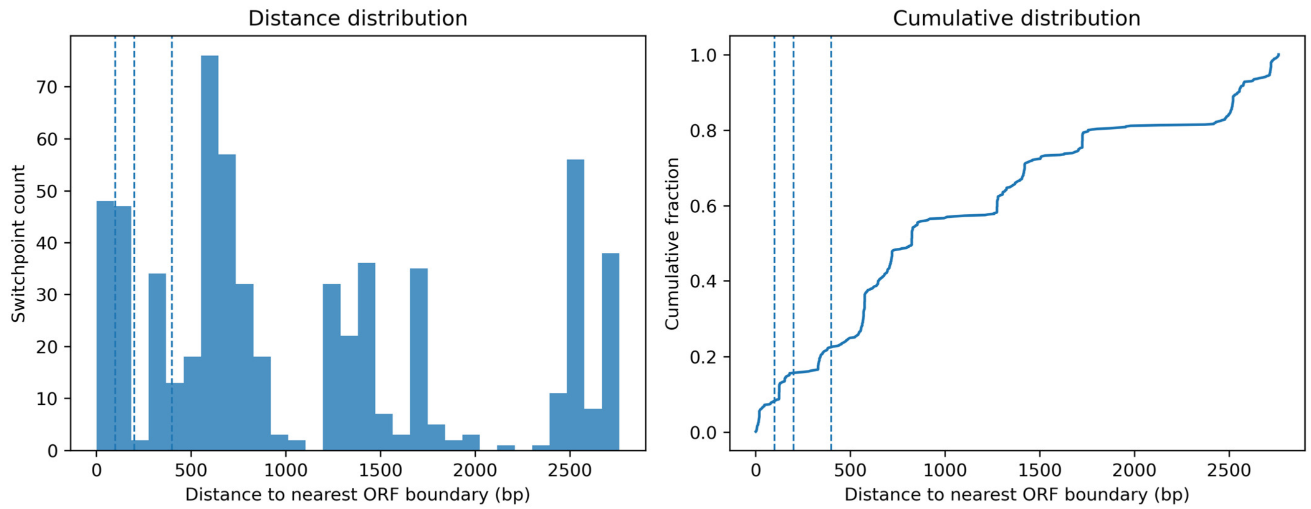

Figure S2. Distribution of switchpoint distances to predicted open reading frame (ORF) boundaries.

(A) Histogram of circular barcode switchpoint distances to the nearest predicted ORF boundary. (B) Cumulative distribution function of the same distances. Dashed vertical lines mark 100-, 200-, and 400-bp boundary neighborhoods evaluated in the threshold-sensitivity analysis. The distributions provide positional context for the significant enrichment detected at 100 and 200 bp and the absence of enrichment at 400 bp in Fig. 3.

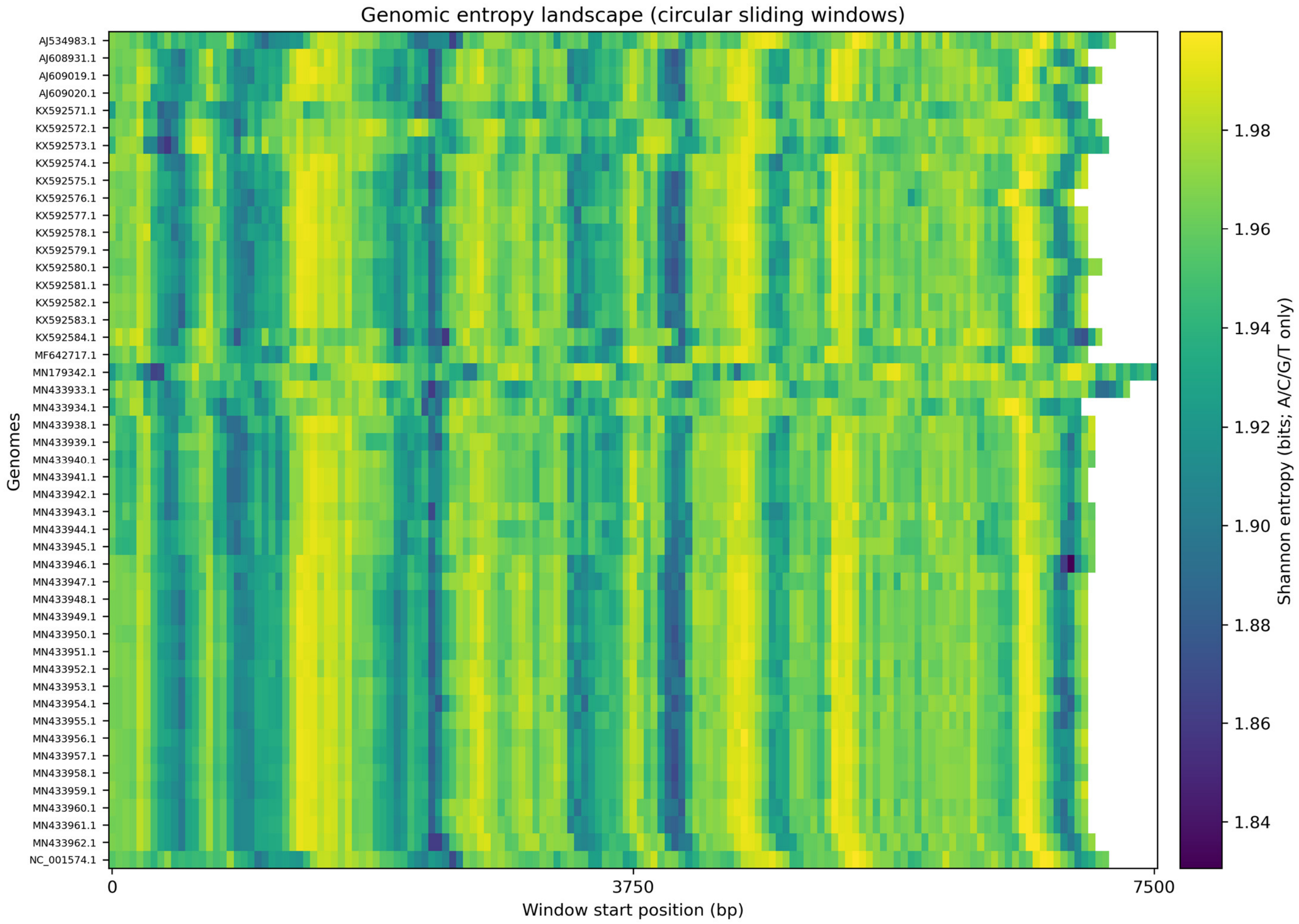

Figure S3. Genome-wide entropy landscapes across cacao swollen shoot virus complex genomes.

Heatmap of circular sliding-window Shannon entropy profiles computed for all 48 genomes (window = 200 bp; step = 50 bp). Entropy was calculated from unambiguous A/C/G/T characters only. Each row represents a genome, and each column represents the window start coordinate along the circular genome. The entropy range is constrained across genomes, providing qualitative context for the mean, variance, dispersion, and tail-statistic comparisons reported in Fig. 7 and Supplementary Data S1.
